# Distinct Associations of BMI and Fatty Acids With DNA Methylation in Fasting and Postprandial States in Men

**DOI:** 10.3389/fgene.2021.665769

**Published:** 2021-05-07

**Authors:** Azucena Pescador-Tapia, Guillermo A. Silva-Martínez, Nicolás Fragoso-Bargas, Dalia Rodríguez-Ríos, Manel Esteller, Sebastian Moran, Silvio Zaina, Gertrud Lund

**Affiliations:** ^1^Department of Genetic Engineering, CINVESTAV Irapuato Unit, Irapuato, Mexico; ^2^Celaya Technological Institute, Celaya, Mexico; ^3^Josep Carreras Leukemia Research Institute (IJC), Barcelona, Spain; ^4^Centro de Investigación Biomédica en Red Cancer (CIBERONC), Madrid, Spain; ^5^Institució Catalana de Recerca i Estudis Avançats (ICREA), Barcelona, Spain; ^6^Physiological Sciences Department, School of Medicine and Health Sciences, University of Barcelona (UB), Barcelona, Spain; ^7^IDIBELL, Barcelona, Spain; ^8^Department of Medical Sciences, Division of Health Sciences, Leon Campus, University of Guanajuato, Leon, Mexico

**Keywords:** prandial state, BMI, fatty acid, DNA methylation, epigenomics

## Abstract

We have previously shown that blood global DNA methylation (DNAm) differs between postprandial state (PS) and fasting state (FS) and is associated with BMI and polyunsaturated fatty acid (PUFA) (negatively and positively, respectively) in 12 metabolically healthy adult Mexican men (AMM cohort) equally distributed among conventional BMI classes. Here, we detailed those associations at CpG dinucleotide level by exploiting the Infinium methylation EPIC array (Illumina). We sought differentially methylated CpG (dmCpG) that were (1) associated with BMI (BMI-dmCpG) and/or fatty acids (FA) (FA-dmCpG) in FS or PS and (2) different across FS and PS within a BMI class. BMI-dmCpG and FA-dmCpG were more numerous in FS compared to PS and largely prandial state-specific. For saturated and monounsaturated FA, dmCpG overlap was higher across than within the respective saturation group. Several BMI- and FA-dmCpG mapped to genes involved in metabolic disease and in some cases matched published experimental data sets. Notably, *SETDB1* and *MTHFS* promoter dmCpG could explain the previously observed associations between global DNAm, PUFA content, and BMI in FS. Surprisingly, overlap between BMI-dmCpG and FA-dmCpG was limited and the respective dmCpG were differentially distributed across functional genomic elements. BMI-dmCpG showed the highest overlap with dmCpG of the saturated FA palmitate, monounsaturated C20:1 and PUFA C20:2. Of these, selected promoter BMI-dmCpG showed opposite associations with palmitate compared to C20:1 and C20:2. As for the comparison between FS and PS within BMI classes, dmCpG were strikingly more abundant and variably methylated in overweight relative to normoweight or obese subjects (∼70–139-fold, respectively). Overweight-associated dmCpG-hosting genes were significantly enriched in targets for E47, SREBP1, and RREB1 transcription factors, which are known players in obesity and lipid homeostasis, but none overlapped with BMI-dmCpG. We show for the first time that the association of BMI and FA with methylation of disease-related genes is distinct in FS and PS and that limited overlap exists between BMI- and FA-dmCpG within and across prandial states. Our study also identifies a transcriptional regulation circuitry in overweight that might contribute to adaptation to that condition or to transition to obesity. Further work is necessary to define the pathophysiological implications of these findings.

## Introduction

The associations of specific DNA methylation (DNAm) profiles with BMI, lipids, and fatty acids (FA) have been documented by observational studies, and in some instances, causality has been established. In the case of BMI, Mendelian randomization approaches have been used in epigenome-wide association studies (EWAS) to assess causality in large cohorts with complex phenotypic stratification. Conflicting results were obtained, as both BMI-to-DNAm and the opposite causality direction were demonstrated (Huan et al., 2019; Sun et al., 2019; Vehmeijer et al., 2020). As for lipid-DNAm causality, circulating lipids cause DNAm and not vice versa (Dekkers et al., 2016). Among FA, arachidonic (AA), oleic (OA), elaidic (EA), and palmitic acid (PA) elicit changes in DNAm in experimental models (Barrès et al., 2009; Hall et al., 2014; Flores-Sierra et al., 2016; Silva-Martínez et al., 2016; González-Becerra et al., 2019). Associations of FA with DNAm have also been uncovered in EWAS and global DNAm studies (Aslibekyan et al., 2014; de la Rocha et al., 2016).

Despite the wealth of data on DNAm and metabolism, the existence of rapid—*i.e.*, within hours—effects of the diet on DNAm in humans is little explored, particularly in the context of obesity. It is conceivable that as part of the normal metabolic adaptation, epigenetic profiles are dynamically reset during fasting to postprandial conditions and that obesity may lead to a loss of resetting ability (Longo and Mattson, 2014). Western-style obesogenic diets might further exacerbate such loss of flexibility. This hypothesis of transition of epigenetic profiles from flexible in pre-obesity to fixed in obesity is conceptually similar to the model that identifies dynamic reprogramming during embryonic development as sensitive to environmental stimuli that can result in the acquisition of stable phenotypic traits in later life (Kwong et al., 2000). Therefore, insights into the epigenetic effects of fasting/postprandial cycles might help in defining novel strategies against obesity.

To address that issue, we previously exploited a cohort of 12 adult Mexican men (AMM cohort) distributed across the three conventional BMI classes. This study design offered several unique advantages. The same subjects underwent fasting (FS) and postprandial states (PS) within a 1–2-week period, and DNAm and individual FA content was measured within a 1–8 h time range. Thus, confounders such as genetic variants and age could be ruled out. Furthermore, AMM was homogeneous with respect to gender, metabolic profiles, and occupational and lifestyle-related factors. The main finding of the original AMM study was an inverse correlation between global DNAm and BMI that was conserved across prandial states. Conversely, polyunsaturated FA (PUFA) were positively associated with DNAm in both prandial states. In particular, eicosapentaenoic acid was positively associated with DNAm in both FS and PS, while AA only showed a positive association with DNAm in PS (de la Rocha et al., 2016).

The present study describes CpG methylation data obtained in the AMM cohort with the Infinium methylation EPIC array (Illumina) (Moran et al., 2016). In particular, we identify differentially methylated CpG (dmCpG) sites associated with BMI and/or FA, within or across FS and PS.

## Materials and Methods

A flow diagram of the study is shown in Supplementary Figure 1.

### Study Participants

The AMM cohort was previously described (de la Rocha et al., 2016). Briefly, AMM consisted of 12 adult male subjects equally distributed across the three conventional BMI classes, normal weight (N), overweight (Ow), and obese (BMI bins 18.5–24.9, 25–29.9, and > 30, respectively). All participants were metabolically healthy (normal blood glucose, triglycerides, cholesterol, and blood pressure), lived in the city of Leon, and were employed as janitors at the University of Guanajuato, Mexico. On one experimental day, subjects who had undergone overnight fasting and abstained from exercise for 48 h received a standard Western-style meal (one each of McDonald’s McChicken, medium-size fries, medium-size Coke) at 10 am, immediately after a venous blood draw which was followed by four additional draws every 2 h (postprandial state or PS). On a different day, the same subjects underwent complete fasting under the same blood draw scheme (fasting state or FS). PS and FS procedures were separated by 7–14 days. All biochemical and molecular determinations were carried out in blood obtained from the same draw. The study was carried out in accordance with the recommendations of the Mexican Government guidelines (NOM-012-SSA3-2012) and the Ethical Committee of the Department of Medical Sciences, University of Guanajuato; the latter also approved the protocol. All subjects gave written informed consent in accordance with the Declaration of Helsinki.

### DNA Methylation Data

The five peripheral blood samples corresponding to each subject were pooled, resulting in 12 samples for each experimental day. Whole-blood DNA was profiled with the Infinium methylation EPIC array (Illumina) according to the manufacturer’s instructions (Moran et al., 2016). Arrays were imaged using BeadArray Reader (Illumina HiSeq 2000) using the standard recommended Illumina scanner setting. The IDAT files were normalized (control normalization) and background corrected using the methylation module (??) available on GenomeStudio (v2011.1) software from Illumina. Probes with the following characteristics were filtered out: SNPs within CpG, probes, or single-base extensions (list compiled by Pidsley et al., 2016); X or Y chromosome probes; probes with detection *p* > 0.01; and those significantly associated with age by Pearson’s or Spearman’s test depending on the normality of distribution (see section “Statistics”). A total of 736,741 probes passed those filters. All genomic coordinates refer to the GRCh37/hg19 assembly. The raw datasets generated for this study were deposited in the GEO repository^1^, ID no. GSE140692.

### Fatty Acid Profiles

Whole-blood total (*i.e.*, free and bound) FA profiles were obtained previously (de la Rocha et al., 2016) and included the following FA: saturated (SFA): myristic (C14:0), PA and stearic acid (C18:0); monounsaturated (MUFA): palmitoleic acid (C16:1n-9), EA, OA, and a mix of C20:1 isomers; and PUFA: linoleic, eicosadienoic (EDA), dihomo-γ-linolenic (DGLA), AA, eicosapentaenoic (C20:5), docosatetraenoic (C22:4n-6), docosapentaenoic (C22:5n-3), and docosahexaenoic (C22:6n-3) acids. C18:3 was determined but excluded as one or more data points could not be determined. Throughout the text, the most widely known FA will be referred to by acronym, the remaining FA by short chemical annotation.

### Statistics

Methylation *M*-values were used for statistics, while the more intuitive β-values were used in data presentation (Du et al., 2010). Probes with one or more missing data points were not considered. The statistical tests applied were dependent on whether data fitted a normal distribution as assessed by the Shapiro–Wilk test, *i.e.*, Pearson’s or Spearman’s test, respectively, in correlations with BMI or FA, and paired *t-*test or Wilcoxon signed-rank test, respectively, in FS versus PS comparisons. Frequencies were compared by the Chi-square test. DNAm was implicitly considered as the dependent variable, based on previous biological evidence (Barrès et al., 2009; Hall et al., 2014; de la Rocha et al., 2016; Silva-Martínez et al., 2016). FA-values were percent normalized. *P*-values were corrected for blood cell composition using the minfi package in R. Nominal (not genome-wide corrected) *p* < 0.05 was considered significant. Gene functional enrichment was tested with the DAVID resource with FDR *p* < 0.05 as significance threshold^2^.

## Results

### Prandial State-Dependent and Independent Associations of DNAm With BMI

**FIGURE 1:**
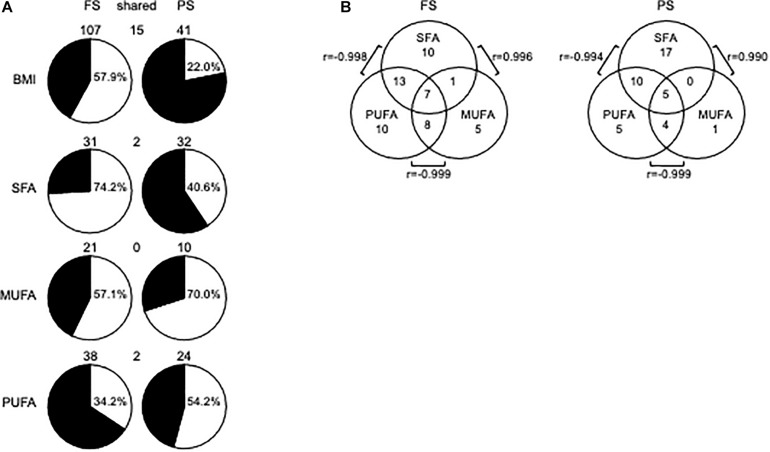
DmCpG in associations with BMI or FA. **(A)** Prandial state-dependent associations of DNA methylation with BMI, SFA, MUFA, and PUFA. In pie charts, white and black indicate hypomethylated (negatively associated) and hypermethylated (positively associated) BMI-dmCpG, SFA-dmCpG, MUFA-dmCpG, and PUFA-dmCpG sets, respectively. Hypomethylated dmCpG percentage is indicated in each pie chart. DmCpG counts are indicated above the respective pie chart. **(B)** Shared dmCpG among SFA, MUFA, and PUFA. *r*-values show correlations between shared dmCpG across saturation groups.

We first sought to identify dmCpG associated with BMI (BMI-CpG). To that end, we performed a correlation analysis between CpG methylation (*M*-values) and BMI across N, Ow, and Ob samples, either in FS or PS. A BMI-dmCpG was called if average Δβ > 0.10 between N and Ob and DNAm M value correlation significance of *p* < 0.05. Although carbohydrates might affect DNAm, we excluded glucose from this and the following association analyses, as glycemia was below the 100-mg/dL normal reference limit in all subjects. We identified a total of 107 and 41 BMI-dmCpG in FS and PS, respectively. Of these, 15 were conserved across prandial states, *i.e.*, 37 and 14% of FS and PS BMI-dmCpG, respectively (Figure 1A and Supplementary Table 1). Notably, the Δβ of BMI-dmCpG conserved between FS and PS were highly correlated (*r* = 0.996, *p* < 10^–6^), indicating high between-array technical consistency. While a slight majority (57.9%) of FS BMI-dmCpG was inversely associated (hypomethylated) with BMI, hypermethylation was predominant (78.0%) of PS BMI-dmCpG (Figure 1A and Supplementary Table 1). That trend resembled the global DNAm trends previously published in FS and in N relative to Ow individuals in PS (de la Rocha et al., 2016).

No significantly enriched pathway or transcription factor target was identified of BMI-dmCpG-harboring genes, and none of BMI-dmCpG overlapped with those reported in EWAS of BMI (Aslibekyan et al., 2015; Demerath et al., 2015; Dhana et al., 2018; Geurts et al., 2018). However, two BMI-dmCpG—cg16733226 in the *CAMK1D* gene body and cg21273765 in the *HIF3A* distal promoter—were associated with BMI in peripheral blood in the Strong Heart Study (Crocker et al., 2020). Focusing on BMI-dmCpG (specific to or shared across prandial states) that were located within promoter regions, we identified single or clustered dmCpG within a number of obesity and metabolism-related genes (Table 1). Examples of the latter were the detoxification gene *GSTM5* in FS and the transcription factor *ZNF718* in PS. Notably, akin to the findings of the Strong Heart Study (Crocker et al., 2020), cg21273765 in *HIF3A* was positively associated with BMI. The direction of association of promoter BMI-dmCpG was similar to the overall trend described previously, *i.e.*, the predominance of negative (hypomethylated) and positive (hypermethylated) associations in FS and PS, respectively (Table 1 and Supplementary Table 1). Of the 15 dmCpG that were shared between FS and PS, three were within promoter regions of the following genes: *MTHFS*, which encodes an enzyme of the folate metabolism pathway; the homeobox-binding *HOPX*; and *SPIRE2*, a player in actin cytoskeleton dynamics and intracellular transport.

**TABLE 1:** Selected relevant promoter BMI-dmCpG.

	Gene	CpG ID	*r*, FS	*r*, *PS*	Function/relevance
FS	*ANKLE2*	cg26577667	0.81		SIRT2 pathway, nuclear envelope assembly (Kaufmann et al., 2016)
	*ASAH1*	cg22061769	−0.83		Ceramide metabolism, NAFLD (Presa et al., 2019)
	*ATP13A4*	cg23159337	0.70		Obesity (Biamino et al., 2016)
	*CCNL1*	cg24725175	0.66		Genetic variants associated with leptin (Kilpeläinen et al., 2016)
	*GSTM5*	cg25210835	−0.63		Detoxification, obesity, NAFLD (Stepanova et al., 2010)
		cg24467349	−0.63		
		cg12858902	−0.58		
		cg23719124	−0.62		
		cg22864244	−0.59		
	*HIF3A*	cg21273765	0.87		Hypermethylated promoter in diabetes and obesity (Zhang et al., 2019; Crocker et al., 2020)
	*HOPX*	cg25456368	−0.65		Cardiomyocyte differentiation (Friedman et al., 2018)
		cg04085076	−0.75		
	*VEGFC*	cg24597353	−0.74		Obesity and insulin resistance (Tinahones et al., 2012)
PS	*DCLK1*	cg05639937		0.67	Inflammation, epithelium repair (Yi et al., 2019)
	*ZNF718*	cg20320494		0.68	Hypermethylated promoter in asthma (Wysocki et al., 2015)
		cg19819404		0.70	
		cg22831726		0.65	
		cg12717203		0.66	
		cg21149944		0.69	
		cg10197305		0.70	
PS and FS	*HOPX*	cg00493422	−0.78	−0.78	Adipocyte differentiation (Hng et al., 2020)
	*MTHFS*	cg04124281	0.69	0.79	Folate metabolism and purine synthesis (Anguera et al., 2003; Misselbeck et al., 2019)
	*SPIRE2*	cg07402062	−0.81	−0.80	Fatty acid synthase regulation (Song et al., 2020)

*NAFLD, nonalcoholic fatty liver disease.*

### Prandial State-Dependent Associations of DNAm With FA

Next, we sought associations between CpG methylation *M*-values and FA grouped by degree of saturation, *i.e.*, SFA, MUFA, and PUFA. Criteria for dmCpG calling were as described above for BMI. Ranking by total dmCpG number was SFA≈PUFA > MUFA, and most dmCpG were prandial state-specific, *i.e.*, on average only 2 and 6% were conserved across prandial states as proportions of FS and PS dmCpG, respectively (Figure 1A and Supplementary Table 2). For SFA-dmCpG and PUFA-dmCpG, the relative proportions of negative and positive associations with DNAm (*i.e.*, hypomethylated and hypermethylated, respectively) were opposite between prandial states, whereas MUFA-dmCpG were mostly hypomethylated in either prandial state (Figure 1A). MUFA-dmCpG profiles mirrored that of BMI-dmCpG in FS, while neither SFA, nor MUFA, nor PUFA-dmCpG profiles mirrored PS BMI-dmCpG. However, SFA and PUFA profiles showed opposite tendencies in either prandial state. Functional analysis revealed that SFA-dmCpG-harboring genes were significantly enriched for targets of nuclear factor kappa B (NFκB), a master regulator of the inflammatory response (FDR < 0.007) (Lawrence, 2009).

As for dmCpG overlap between FA saturation groups, 53% of SFA-dmCpG were specific to that group. Conversely, the majority of MUFA-dmCpG and PUFA-dmCpG were shared with one and/or the other saturation group (Figure 1B). For example, 90% and 79% of MUFA-dmCpG and PUFA-dmCpG, respectively, overlapped with other saturation groups. In particular, in either prandial state highest and lowest overlap was between SFA-dmCpG and PUFA-dmCpG and SFA-dmCpG and MUFA-dmCpG, respectively. Notably, for the former the direction of association was negative, while the latter showed a positive association. A total of seven and five dmCpG overlapped in all three saturation groups in FS and PS, respectively. Of these, two were located within regulatory regions, *i.e*., in *SETDB1* in FS and *LASS3* in PS. In either gene, CpG methylation correlated negatively with SFA and MUFA but positively with PUFA, akin to the previously published associations between FA saturation and global DNAm (de la Rocha et al., 2016).

To understand whether dmCpG of a given saturation group were representative of all individual FA-dmCpG within that group, we probed for associations between CpG methylation values and individual SFA, MUFA, and PUFA (three, four, and eight FA, respectively). Individual FA-dmCpG count ranged between 2 and 58 in FS and between 0 and 32 in PS, with EDA and C16:1 being associated with most and least dmCpG, respectively. PA, C20:1, EDA, and EPA were associated with most dmCpG within SFA, MUFA, n-6, and n-3 PUFA, respectively (Supplementary Table 3 and Supplementary Figure 2). As observed for BMI and FA saturation groups, individual FA-dmCpG were significantly more abundant in FS than in PS (average FS/PS ratio = 3.5; *p* = 0.009), with only a minority shared between prandial states, *i.e.*, 5 and 12% in FS and PS, respectively. With a few exceptions (C14:0, C18:0, AA, C22:4n-6), individual FA showed opposite associations with CpG methylation in FS and PS (Supplementary Figure 2). Within each saturation group, dmCpG profiles were consistent across individual PUFA with the exception of AA but heterogeneous across individual SFA and MUFA. However, the most abundant individual FA in blood of each group based on percentage (OA, PA, and linoleic acid, respectively) largely mirrored the overall methylation associations of each saturation group, with the exception of OA in PS (compare Figure 1A and Supplementary Figure 2). In FS, we corroborated the previously documented tendency of EPA and EA to elicit hypermethylation and the hypomethylating effect of OA (de la Rocha et al., 2016; Flores-Sierra et al., 2016). Notably, the limited dmCpG overlap between the MUFA isomers OA and EA is in line with their previously reported divergent effects on DNAm (Flores-Sierra et al., 2016). Indeed, the paucity of shared dmCpG within a saturation group compared to dmCpG shared among saturation group was particularly obvious for SFA-dmCpG and MUFA-dmCpG, *i.e.*, overlap within those saturation groups was on average 3% (Supplementary Figure 3 and Supplementary Table 3) but ∼4–5-fold higher across saturation groups (15 and 12%, respectively; *p* < 0.01 in either case, Mann–Whitney *U*-test) (Supplementary Table 4). PUFA-dmCpG followed a different trend, as overlap either within PUFA or with SFA/MUFA groups was comparatively high (27 and 14%, respectively; *p* > 0.05).

No significant functional enrichment was detected of individual FA-dmCpG-harboring genes, and none overlapped with loci associated with n-3 PUFA intake in EWAS (Aslibekyan et al., 2014). However, several FA-dmCpG mapped within promoter regions of pathobiologically relevant genes. All were prandial state-specific, with the exception of dmCpG in *DTX2* and *LCLAT1*, and several were shared across individual FA from different saturation groups (Table 2). Furthermore, we uncovered some general patterns with respect to the direction of association across individual FA from distinct saturation groups: (1) an opposite direction between SFA PA-dmCpG on the one hand, and MUFA C20:1-dmCpG and individual PUFA-dmCpG on the other, and (2) a concordance between SFA PA-dmCpG and MUFA OA-dmCpG. For each of these FA, the described associations mirrored their overall dmCpG profiles (Supplementary Figure 2). The most notable prandial state-specific profile was a 10-dmCpG region in the 5′ region of *LHX6*, encoding a pleiotropic DNA-binding protein that controls craniofacial development (Zhou et al., 2015; Table 2). These 10 dmCpG (collectively referred to as dmLHX6) were specific for FS C20:1-dmCpG, EDA-dmCpG, and DGLA-dmCpG and overlapped with the binding sites of the transcriptional regulator EZH2 (chromosome 9:124,987,559–124,990,236), which is implicated in adipogenesis, among other phenomena (Wang et al., 2010). Interestingly, three dmLHX6 CpG—cg04282082, cg13571460, and cg21469772—were hypermethylated, albeit only by 3–5%, in human monocytes stimulated with increasing AA doses in the 1–10-μM range (Silva-Martínez et al., 2016). The PUFA AA was associated with dmLXH6, although significance was lost after correcting for cell type composition. Therefore, the associations of dmLHX6 with C20:1, EDA, and DGLA are likely causal. Another notable case are dmCpG within *HIVEP3* or Schnurri-3, encoding a ligand of the kappaB enhancer motif that regulates the inflammatory response, which were positively associated with PA and OA but negatively with selected PUFA (Hicar et al., 2001; Oukka et al., 2002; Supplementary Table 3, Table 2). Interestingly, we previously found that *HIVEP3* cg23762517 and cg26038582 methylation correlated inversely with expression in THP-1 cells stimulated with OA in the 1–10-μM range (*r* = −0.61; Affymetrix Human Exon 1.0 ST data and 450K data), thus mirroring the positive association of OA with *HIVEP3* promoter dmCpG observed here (Silva-Martínez et al., 2016). Additional dmCpG in regulatory regions of pathobiologically relevant genes included PA-dmCpG within the *ACBD5* that is involved in long-chain FA metabolism (Ferdinandusse et al., 2017); CD34, an adipocyte progenitor marker (Hui et al., 2007); and CDH18 that hosts variants associated with the metabolic syndrome (Zhang et al., 2013). Importantly, the previously documented associations of FA with *PDK4* and *HDAC4* regulatory regions in AMM (de la Rocha et al., 2016) showed the same tendency as previously observed (de la Rocha et al., 2016), albeit none of the corresponding dmCpG passed the calling filter. Taken together, the data indicate that associations of FA with DNAm are better captured by analyzing individual FA rather than FA grouped by degree of saturation, in particular of SFA and MUFA. Also, experimental data sets support a causal role for FA at least in the case of *LHX6* and *HIVEP3* promoter dmCpG.

**TABLE 2:** Selected 5′ region dmCpG associated with multiple FA.

Gene, CpG ID	FA, association *r*						Relevance
	SFA		MUFA		PUFA		
		
	FS	PS	FS	PS	FS	PS	
*CBLN1*							Highly expressed in adipose tissue, lipid-binding activity (Ye et al., 2020)
cg09792926		PA, 0.65		OA, 0.64 C20:1, −0.63		LA, −0.62 EDA, −0.73 DGLA, −0.69	
*CCAR1*							Transcription regulation (Kim et al., 2008)
cg13515095		C14:0, −0.75 PA, 0.64				EDA, −0.78 DGLA, −0.61 EPA, −0.59 C22:5n-3, −0.61	
*DTX2*							Notch signaling (Kishi et al., 2001)
cg03509467			C20:1, −063	EA, −0.66 C20:1, −0.62	EDA, −0.62 DHA, −0.67	EDA, −0.60 DGLA, −0.75	
cg06619150		PA, 0.59	C20:1, −0.59			AA, −0.71 DHA, −0.64	
*HIVEP3*							Inflammation (Hicar et al., 2001; Oukka et al., 2002)
cg11769885					EPA, −0.66		
cg23762517	PA, 0.59	PA, 0.58	OA, 0.60		EPA, −0.73	EDA, −0.65	
cg26038582	PA, 0.63		OA, 0.65		EPA, −0.73		
*LCLAT1*							Cardiac homeostasis (Bravo-San Pedro et al., 2017)
cg15652532			C20:1, 0.70		EDA, 0.60	EDA, 0.67 DGLA, 0.69	
*LHX6*							Craniofacial development (Zhou et al., 2015)
cg00142257			C20:1, 0.64		EDA, 0.58		
cg03363289			C20:1, 0.60				
cg04282082			C20:1, 0.69		EDA, 0.68		
cg05037505			C20:1, 0.84		EDA, 0.78 DGLA, 0.61		
cg05136264			C20:1, 0.65		EDA, 0.62		
cg11479503			C20:1, 0.70		EDA, 0.71 C22:4n-6, 0.66 DHA, 0.87		
cg13571460			C20:1, 0.74		EDA, 0.78 C22:4n-6, 0.73 DHA, 0.85		
cg13862711			C20:1, 0.67		EDA, 0.66		
cg15124400			C20:1, 0.80		EDA, 0.76 C22:4n-6, 0.61		
cg21469772			C20:1, 0.70		EDA, 0.74 C22:4n-6, 0.64		
*MIR487B*							Ischemia, vascular remodeling (Nossent et al., 2013; Van Der Kwast et al., 2018)
cg18863119		PA, −0.71		C20:1, 0.58		EDA, 0.76 DGLA, 0.63	
*MIR539*							Heart failure (Muthusamy et al., 2014)
cg14667980		PA, −0.64		C20:1, 0.58		EDA, 0.68 DGLA, 0.62	
*POU2AF1* (*OCAB*)							Brown fat tissue accumulation, insulin resistance (Carter et al., 2018)
cg24049888				C20:1, 0.76		EDA, 0.68 DGLA, 0.61	
*SETDB1*							Regulation of chromatin, lipid storage (Zheng et al., 2018)
cg10589385	PA, −0.66				AA, 0.64 EPA, 0.62 C22:4n-6, 0.79		

### Comparison Between BMI and Individual FA-Associated DNAm Profiles

**FIGURE 2:**
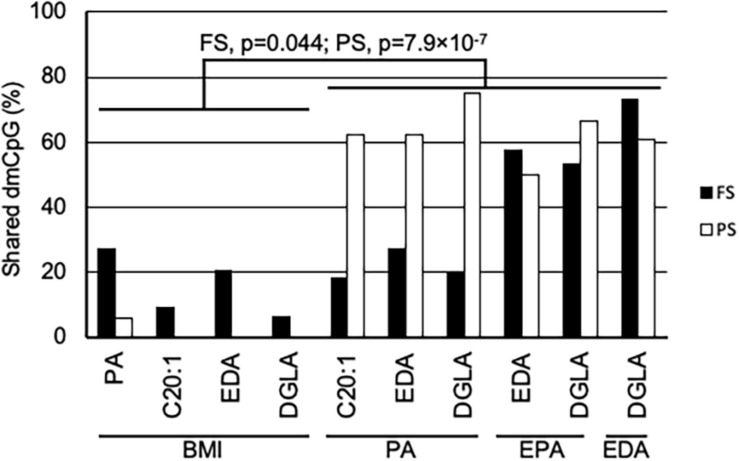
Shared dmCpG between BMI and FA. Percentage of shared dmCpG between BMI and FA, or among FA in FS (solid bars) and PS (open bars). Percentages were calculated of FA dmCpG counts in BMI-FA comparisons, or of the lowest dmCpG counts in FA-FA comparisons. FS and PS, fasting and postprandial state, respectively. Significance of differences between the percentage of dmCpG shared by BMI and FA and shared by FA is shown in FS and PS (Mann–Whitney *U*-test).

A general comparison of the associations with CpG methylation in FS and PS identified a number of patterns. Firstly, prandial state-associated BMI-dmCpG were ∼2–35-fold more abundant compared to individual FA-dmCpG. Shared dmCpG between FS and PS as percentage of PS dmCpG followed a similar trend (BMI: 36.6%; all FA: average 10.5%) (Figure 1A and Supplementary Figure 2). Secondly, dmCpG tended to be shared between BMI and FA more frequently in FS compared to PS. For example, the SFA PA-dmCpG, MUFA C20:-dmCpG, PUFA EDA-dmCpG, and DGLA-dmCpG were shared between BMI and FA on average ∼10-fold more frequently in FS compared to PS (16.0% and 1.5% of the corresponding FA-dmCpG, respectively; *p* = 1.9 × 10^–11^, Chi-square test) (Figure 2). Of note, these particular FA were chosen based on an arbitrary threshold of > 15 dmCpG in each prandial state for statistical robustness. Also, pathobiological relevance has also clearly been established for some of those FA: PA is a proinflammatory FA that induces DNA hypermethylation in cultured cells (Barrès et al., 2009; Hall et al., 2014), and EDA can be converted to AA and DGLA, two precursors of inflammation modulators (Brenner, 1974; Huang et al., 2011). Thirdly, dmCpG were shared to a lesser extent between BMI and FA than across FA in FS (16.0% and 41.6%, respectively; *p* = 0.044, Mann–Whitney *U*-test) and even more markedly so in PS (1.5% and 63.1%, respectively; *p* = 7.9 × 10^–7^) (Figure 2). This trend was also observed when all FA were considered in PS (BMI-dmCpG and FA-dmCpG overlap, 7.5%; overlap across FA-dmCpG, 18.2%; *p* = 0.029) but not in FS (Supplementary Table 4). A list of overlapping BMI-dmCpG and FA-dmCpG located within promoter regions is shown in Table 3. Of note, the directions of association with BMI were discordant with the MUFA C20:1 and the PUFA EDA and DGLA, but concordant with the SFA PA. A further notable example is six *ZNF718* dmCpG positively associated with BMI (Table 1) but negatively with linoleic acid (Supplementary Table 3).

**TABLE 3:** Promoter dmCpG associated with BMI and FA.

Gene, CpG ID	Association *r*										Relevance
	BMI		PA		C20:1		EDA		DGLA		
	FS	PS	FS	PS	FS	PS	FS	PS	FS	PS	
*CRISP2*											Associated with atherosclerosis (Istas et al., 2017)
cg01076129	−0.85		−0.76		0.62		0.80				
cg14997592							0.61		0.64		
cg15509430	−0.73		−0.59				0.70				
cg25390787							0.68		0.62		
cg26715042	−0.70		−0.83				0.76		0.69		
cg12440062		−0.79									
*ESPNL*											Marker of alcohol exposure (Clark et al., 2018)
cg15871206				0.61		−0.69				−0.66	
cg19391697		0.60									
*ZNF260*											Elevated in hypertension (Debrus et al., 2005)
cg06920214	−0.82		−0.69		0.62		0.71				

To further compare prandial state-related features of CpG methylation associations with BMI and FA, we determined the genomic distributions of dmCpG relative to gene compartments and CpG islands. In the latter distribution, the genome is divided into CpG islands, shores (2-kb regions adjacent to either side of a CpG island), shelves (2-kb regions adjacent to shores, on either side of a CpG island), and open sea (rest of the genome). CpG islands are mostly unmethylated regions that can undergo hypermethylation in aging and atherosclerosis, while aberrant methylation of shores has been linked to cancer (Issa et al., 1994; Irizarry et al., 2009; Castillo-Díaz et al., 2010). With respect to individual FA, we restricted the analysis to the aforementioned FA that had > 15 dmCpG in either prandial state (*i.e.*, PA, C20:1, EDA, and DGLA). Supplementary Figure 4 shows a summary of dmCpG distributions and their comparisons with either the 736,741 Infinium methylation EPIC array reference probes, between FS and PS, or among dmCpG sets. In either prandial state, BMI-dmCpG distribution relative to gene compartments or CpG islands was not significantly different from the distribution of the array reference probes. Conversely, all FA-dmCpG—with the exception of FS C20:1-dmCpG—differed from reference probe distribution in the majority of cases, with stronger significance in PS relative to FS. FA-dmCpG and BMI-dmCpG distribution across gene compartments was more similar in FS than in PS. Likewise, only FA-dmCpG distribution differed significantly across prandial states. Also, across FA-dmCpG, 5′-regulatory regions were more frequent in PS compared to FS (*p* < 10^–6^), but CpG islands were less represented (∼1.4–1.6-fold; *p* < 0.01). In all cases, CpG islands were mostly outside promoters (gene bodies and intergenic).

Next, we asked whether dmCpG were differentially distributed among quartiles of β-values in N samples. The rationale for this analysis was that regulatory regions across the genome display characteristic DNAm levels. In particular, partially methylated regions are prone to dynamic changes in DNAm in response to regulatory DNA-binding proteins and undergo global changes in cancer (Stadler et al., 2011; Berman et al., 2012). In both FS and PS, BMI-dmCpG were most abundant in N sample upper-intermediate β-values (0.5–0.75) and did not significantly differ between FS and PS (Supplementary Figure 5). Interestingly, 15 out of 16 CpG island BMI-dmCpG had β > 0.25 (average: 0.57; range: 0.29–0.76) in FS N samples, and the majority (11 out of 16) underwent further hypermethylation with increasing BMI. In PS, that pattern was less obvious, as ∼50% of CpG island dmCpG were highly methylated in N samples. With respect to specific FA, PA-dmCpG and EDA-dmCpG were significantly different between FS and PS, tending to be shifted toward intermediate-low β in PS (*p* = 1.1 × 10^–5^ and *p* = 7.8 × 10^–9^, respectively).

### The Prandial State Exerts BMI Class-Specific Effects on DNAm Profiles

**FIGURE 3:**
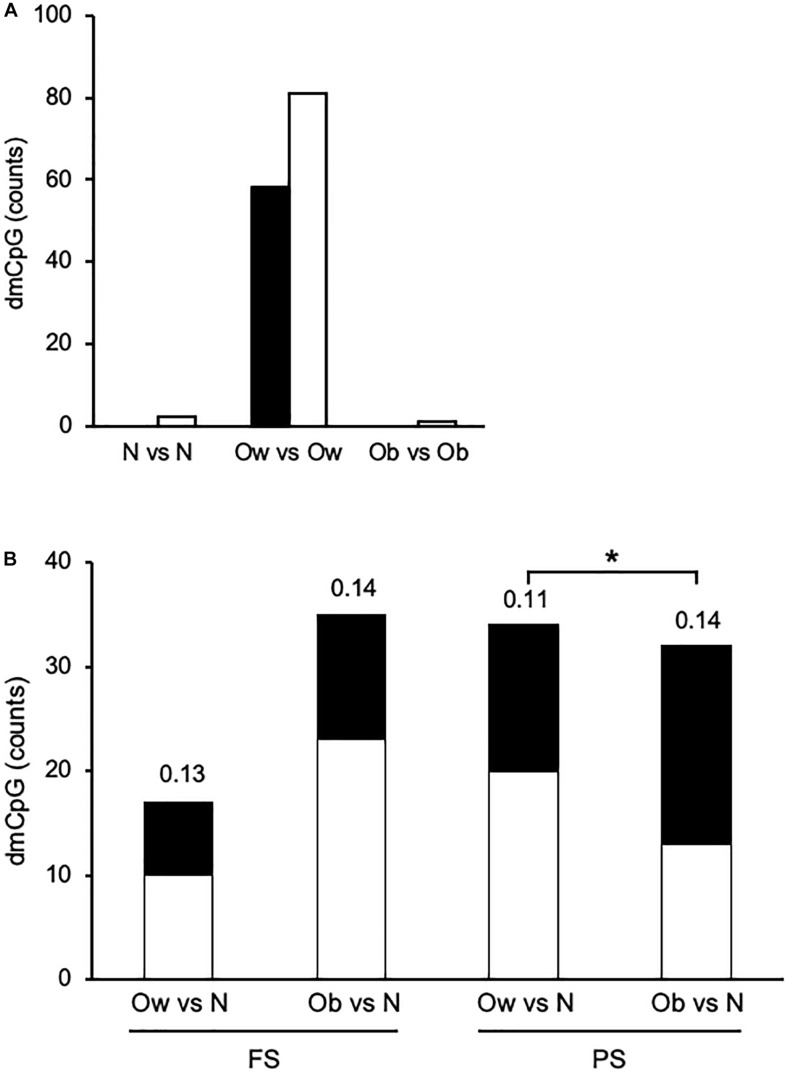
Overview of prandial state-induced and BMI category-associated DNA methylation changes. **(A)** Within each BMI class in PS relative to FS. **(B)** Across BMI classes. Solid and open bars, hypermethylated and hypomethylated, respectively. The number above each bar indicates the average absolute Δβ. N, Ow, and Ob, normoweight, overweight, and obese, respectively. *, *p* < 0.05. *t*-test between *M*-values.

Finally, we sought differential DNAm between FS and PS. To that end, we compared CpG methylation profiles within each of the three BMI classes—*i.e.*, N in FS against N in PS, and the corresponding comparisons for Ow and Ob. In this case, dmCpG were called when the difference between average β > 0.10 and *M*-value paired comparison *p* < 0.05. Strikingly, the comparison between Ow yielded a total of 139 dmCpG (Ow-dmCpG), whereas the comparisons between N and between Ob yielded only two and one dmCpG, respectively (Figure 3A). The dmCpG list is provided in Supplementary Table 5. This large difference in dmCpG counts could result either from peculiar characteristics of the overall Ow dataset or from nonbiological factors such as sample handling or microarray technical issues. Yet, the comparison between Ow and N or between Ob and N in either FS or PS yielded predictable profiles, *i.e.*, dmCpG counts were ∼2-fold larger in the Ob-N comparison relative to Ow-N in FS and were similar in the two comparisons in PS—*i.e.*, no excess dmCpG count was observed in Ow (Figure 3B). DmCpG were identified by the same filtering criteria as above (*p* < 0.05 in M value comparisons, Δβ > 0.10 between Ow or Ob and N). Also, the extent of differential methylation as measured by average Δβ was similar across comparisons and indeed lower in the Ow-N comparison relative to Ob-N in PS (0.11 ± 0.02 and 0.14 ± 0.06, respectively; *p* = 0.016). Furthermore, BMI was not more variable in Ow: SD expressed as percentage of average increased with BMI and was 1.4, 5.7, and 8.8 in N, Ow, and Ob, respectively, in FS. The same trend was observed in PS: 4.6, 5.7, and 9.1, respectively. Furthermore, as a possible explanation for the distinct behavior of Ow-dmCpG, we sought associations of the 139 Ow-dmCpG with all available metabolic (triglycerides, cholesterol, lipoproteins, glucose), somatometric (height, waist and hip circumference), and vascular (systolic and diastolic blood pressure) parameters. No significant association was detected in any case. We conclude that the observed difference between FS and PS Ow is specifically imposed by the combination of prandial state and BMI class, although the underlying mechanism is unclear. Notably, Ow-dmCpG-hosting genes were significantly enriched in targets of known transcription factors involved in lipid metabolism and obesity (*p* < 0.05, FDR; Supplementary Table 6). No overlap of Ow-dmCpG-hosting genes with any of the published studies indicated above was observed.

Ow-dmCpG showed a slight trend toward hypomethylation in PS relative to FS (81 of 139 Ow-dmCpG or 58.3%). Ow-dmCpG genomic distribution relative to gene compartments or CpG islands was significantly skewed in favor of promoters and first exons (∼1.8-fold enrichment, p = 0.006; Chi-square test) and CpG islands (∼2.3-fold, *p* = 9.7 × 10^–8^), where enrichment was highest of any dmCpG set, compared to the distribution of the 736,741 Infinium methylation EPIC array reference probes (Supplementary Figure 4). That enrichment was contributed to by the Ow-dmCpG fraction that was hypermethylated in PS, which was ∼4.5-fold enriched in CpG islands (*p* = 8.7 × 10^–67^) and ∼5-fold in promoters, while ∼3-fold and ∼21-fold depleted in gene bodies and intergenic regions, respectively (*p* = 2.9 × 10^–53^), compared with the hypomethylated fraction. Ow-dmCpG distribution was significantly different from BMI-dmCpG and any FA-dmCpG, either in FS or PS. Distribution across FS β quartiles was highly skewed toward unmethylated or very low methylation regions for hypermethylated Ow-dmCpG but peaked in the middle-high methylation β quartile (0.5-0.75) in the case of the hypomethylated set (Supplementary Figure 5). Since 79.1% of promoter/5′UTR/first exon and 82.4% of CpG island Ow-dmCpG had β < 0.5 in FS, we conclude that hypermethylation of the promoter and CpG island with low baseline methylation was a distinct feature of Ow-dmCpG. No shared dmCpG among the three PS-to-FS comparisons, with BMI or FA, was observed.

**FIGURE 4:**
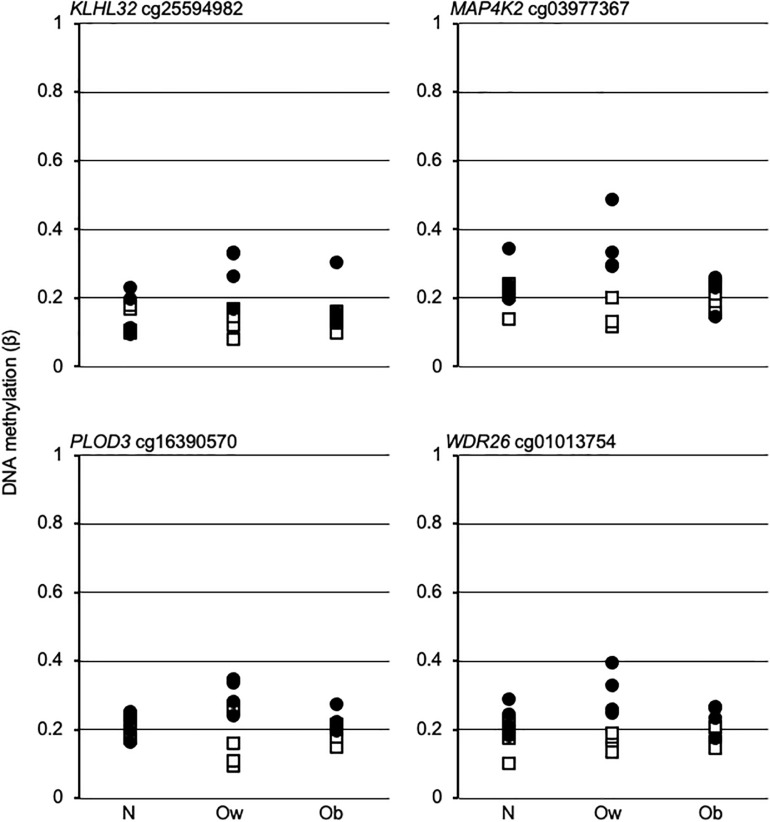
DNA methylation trend in fasting and postprandial states across BMI classes. Open squares and solid circles, fasting and postprandial state, respectively. Gene name and EPIC array Ow-dmCpG ID are indicated above each graph. N, Ow, and Ob, normal weight, overweight and obese, respectively.

The above data suggest that the postprandial dynamic remodeling of DNAm in Ow might establish Ob-specific epigenetic profiles upon transition from overweight to obesity. To assess that hypothesis, we examined the β-values of four promoter dmCpG mapping to relevant genes and with the highest absolute Δβ between PS and FS Ow, across BMI classes. The data indicated a larger variability in Ow compared to N or Ob. However, no Ob-specific profile could be observed, as N and Ob profiles were indistinguishable (Figure 4).

## Discussion

We reveal for the first time two distinct associations among prandial state, BMI, FA, and CpG methylation in human peripheral blood. Firstly, the associations of CpG methylation with BMI and FA differ in fasting and postprandial states, in terms of number and identity of targeted CpG, and direction of CpG methylation change. Also, BMI-dmCpG and most FA-dmCpG are more numerous in FS relative to PS. Secondly, the number of dmCpG between prandial states within the same BMI class is dramatically different among BMI classes.

Comparisons of associations with BMI and with FA reveal that BMI-dmCpG are more abundant than FA-dmCpG, when either prandial state-specific or shared between prandial states dmCpG are considered. These observations underline the relevance of the BMI, either as causal factor or covariable, for the epigenome. The paucity of shared dmCpG also indicates that associations of BMI and FA are mechanistically distinct. This conclusion is reinforced by the observation that BMI-dmCpG and FA-dmCpG map to genomic regions with distinct baseline methylation levels and positions relative to CpG islands or gene compartments: BMI-dmCpG are not significantly enriched in any genomic context and have mostly intermediate–high baseline methylation level. Conversely, PA-dmCpG and EDA-dmCpG are in distinct baseline methylation level bins in FS and PS, and FA-dmCpG are generally differentially distributed relative to CpG islands and gene compartments in FS and PS. Also, CpG island FS BMI-dmCpG, C20:1-dmCpG, and EDA-dmCpG are highly methylated and undergo further hypermethylation with increasing BMI or FA. This pattern is unusual, as CpG islands are mostly unmethylated or methylated at low levels throughout the genome (Bird et al., 1985; Edwards et al., 2017). Corresponding PS CpG island dmCpG do not show such polarized features.

Our study design strongly suggests causality of the prandial state with respect to DNAm, possibly via interaction with genetics or environmental/dietary variables. Yet, the mechanism underlying prandial state-specific associations of DNAm with BMI or FA can only be speculated. BMI and FA might differentially affect DNAm depending on the postprandial or fasting metabolic conditions or might be inert covariates of the causal factor. Our CpG filtering method rules out diet-dependent blood cell composition effects. Also, it is unlikely that results were affected by statistical artifacts due to the difference in FA pool composition between FS and PS (de la Rocha et al., 2016). Specifically, the paucity of associations in PS could be due to more homogeneous distributions in that group: yet, taking PA as example, correlation r is in the −0.85–+0.85 range in FS and PS. Also, PA tends to increase with BMI and actually does so more markedly in PS than in FS (105.0% and 57.9% increase in Ob compared to N, respectively). Additionally, dmCpG were often specific to FS or PS. In fact, FS and PS shared relatively few FA-associated DNAm profiles, and a number of dmCpG switched the direction of associations depending on the nature of the individual FA, within FS or PS. Another consideration is that the directions of association were consistent for most PUFA on the one hand, and for BMI, OA, and PA on the other, in accordance with the accepted pathophysiological properties respectively assigned to PUFA and SFA. Notably, the position of the MUFA OA is ambiguous, as is its association with disease risk (Steffen et al., 2018).

Distinct patterns emerged also at the individual CpG level. For some dmCpG, causal effects of BMI or FA on DNAm are strongly supported either by direct mechanistic data such as dmLHX6 profiles and *HIVEP3* expression in cultured cells stimulated with AA or OA or by independent functional and gene expression studies of promoter regions of *LHX6*, *ASAH1*, *MIR487B*, and *HIF3A* as follows: (1) LHX6 controls embryonic skeletal development, and the relevance of dmLHX6 is supported by the known involvement of PUFA and their metabolites in craniofacial development in animal models (Greene and Garbarino, 1984; Kay et al., 1988). DmLHX6 are uniquely associated with three C20 FA which are precursors or intermediates of the same metabolic pathway, thus pointing to specific biological activity. (2) The negative association of *ASAH1* promoter methylation with BMI is in accordance with higher *ASAH1* expression in a mouse NAFLD model (Presa et al., 2019). (3) Hypomethylation of the *MIR487B* promoter with increasing PA, but hypermethylation with increasing C20:1, EDA, and DGLA, is in agreement with its previously documented adverse effect of *MIR487B* on insulin signaling and upregulation in hypertension *in vivo* in a rat model, validated in human cultured cells (Nossent et al., 2013). 4) Three independent studies including ours consistently point to the association of *HIF3A* promoter hypermethylation with adverse metabolic outcomes such as obesity and insulin resistance, in omentum of gestational diabetes mellitus patients and in human peripheral blood cells analyzed by EWAS (Zhang et al., 2019; Crocker et al., 2020). Another relevant finding is differential methylation of *CRISP2.* The data are more difficult to interpret in this case, as hypermethylation of the *CRISP2* promoter CpG cg26715042 in peripheral blood cells is associated with atherosclerosis, but the opposite association is observed here with BMI (Istas et al., 2017). It is possible that our data represent compensatory responses to the establishment of obesity in metabolically normal subjects, whereas the literature points to phenomena occurring in metabolically heterogeneous obese samples. A further *caveat* is that previous studies were based on a variety of biological models and may not be readily comparable with the approach present here. It is therefore not surprising that our findings did not replicate any of the published BMI or FA EWAS, with the exception of the Strong Heart Study (Crocker et al., 2020). A likely reason for those discrepancies is the specific characteristics of our cohort, particularly its homogeneity in terms of gender, age, race, occupational and environmental exposure, and absence of any complication of obesity. Taken together, several considerations suggest that our findings are biologically plausible, despite the two main limitations of this study: small sample size and lack of information on dietary intake prior to sampling.

In addition to prandial state-specific profiles, our study uncovered BMI-associated DNAm profiles that overlapped between FS and PS and thus are testable candidate causal loci of later complications such as metabolic syndrome, CVD, diabetes, and fatty liver disease. Of particular interest was the positive association between promoter methylation of *MTHFS* and BMI in FS and PS. Notably, upregulation of *MTHFS* lowers cellular folate, an essential cofactor for methylation reactions, which could potentially explain the inverse correlation between global DNAm and BMI previously reported in FS and PS (Anguera et al., 2003; de la Rocha et al., 2016). In that study, we also showed that in both prandial states SFA and MUFA were inversely associated with global DNAm, while the opposite was true for PUFA. Interestingly, *SETDB1* promoter methylation mirrored the above-described associations in FS. SETDB1 is an important regulator of lipid storage (Zheng et al., 2018), in addition to being the most important driver of transposon methylation in embryonic stem cells and somatic tissues. SETDB1 is upregulated during early embryogenesis when the mammalian genome undergoes a global hypomethylation event (Fukuda and Shinkai, 2020). This might suggest that fasting-induced SETBD1 promoter hypomethylation represents a compensatory mechanism for transposon silencing during fasting-induced BMI-dependent global DNA hypomethylation. Furthermore, the ceramide synthase *LASS3* promoter methylation may be predicted to decrease global DNAm given the recent finding of the DNA hypermethylating potential of ceramides in PS (Castro et al., 2019).

Also, the comparison of our previous global DNAm survey in AMM and the present study reveals differences in association directions for BMI and FA, particularly in PS. These discrepancies suggest a markedly different behavior of repeated and non-repeated genomic sequences.

Lastly, we demonstrate that the prandial state affects CpG methylation in a BMI class-dependent fashion. Surprisingly, dmCpG between PS and FS are vastly more abundant in the Ow group compared to N and Ob, in addition to showing relatively high methylation variability. Thus, the combination of Ow and prandial state generates dmCpG, echoing evidence that BMI causes DNAm (Sun et al., 2019). Yet, no overlap with BMI-dmCpG exists. In addition, Ow-dmCpG were enriched in CpG islands and promoters with low baseline methylation level that undergo hypermethylation in PS compared to FS, a distinct pattern in comparison with BMI-dmCpG (see above). The dynamic epigenetic profiles of Ow likely represent the initial drift during the transition to obesity, or an adaptation to overweight. A testable hypothesis is that the intrinsic Ow epigenetic dynamicity and prandium-induced differential methylation transiently modifies the expression of critical genes, thus setting a transcriptional program in motion that proceeds autonomously once obesity is reached. Ow-dmCpG mapped to a number of genes involved in obesity and metabolism. Notable examples were *MAP4K2* or *GCK*, close to an SNP associated with uric acid and a candidate player in inflammation (Chuang et al., 2016; Lee et al., 2018); *IRX2*, associated with BMI in women and expressed in an adipose tissue depot-dependent fashion (Karastergiou et al., 2013; Ng et al., 2017)—IRX2 promotes cellular differentiation in leukemia and could therefore exert currently unappreciated obesity-related effects in blood cells, for instance by altering the leukocyte repertoire (Scalea et al., 2020); *KLF3*, a driver of diet-induced obesity in mice which could act by promoting the expression of the adipose browning factor in selected white blood cells (Bell-Anderson et al., 2013; Knights et al., 2020); *RREB1*, promoting adipose tissue browning (Brunmeir et al., 2016)—the RREB1-altered expression in peripheral blood is associated with pediatric obesity (Plaza-Florido et al., 2020); *KLHL32*, associated with BMI in a blood-based genome-wide association study (Monda et al., 2013); *PLOD3* (aka *LH3*), involved in adiponectin production (Zhang et al., 2015)—peripheral blood *PLOD3* expression is altered in obstructive sleep apnea, a condition linked to obesity (Perry et al., 2013); and *HVCN1*, a driver of reactive oxygen species production in B cells (Capasso et al., 2010). The most significant enrichment was of targets for E47, an activator of adiponectin expression (Doran et al., 2008). E47 regulates hematopoiesis, and its expression in blood cells could therefore determine to establish obesity-related lineages (Meyer et al., 2020). Other relevant target enrichments were for RREB1, which is also encoded by an Ow-dmCpG-hosting gene, and for SREBP1, a partner of E47 in adiponectin expression activation and an extensively studied regulator of FA metabolism (Doran et al., 2008; Jump, 2011). Methylation of selected *SREBP1* CpG is also associated with adiposity (Justice et al., 2020). Also, SREBP1 has been implicated in establishing a proatherogenic lipid profile in monocytes (Bowman et al., 2020). In summary, several of the indicated genes have well-characterized functions in adipocytes, but evidence for regulation of blood cell homeostasis supports our differential methylation data. Based on transcription factor-binding site enrichment data, E47/SREBP and RREB1 might be master regulators of Ow-specific transcriptional profiles. In particular, our data suggest a loop in RREB1 signaling, with reduced RREB1–target gene interaction, consistent with the enrichment in hypermethylated promoters, secondary to an increase in *RREB1* promoter methylation induced by an unidentified mechanism in PS. The functional roles for RREB1 and E47 are supported by their respective documented beneficial effects on adipocyte browning and adiponectin expression (Doran et al., 2008; Brunmeir et al., 2016). At any rate, further studies with a larger sample size are necessary for confirming the observed Ow epigenetic dynamicity.

## Data Availability Statement

The datasets presented in this study can be found in online repositories. The names of the repository/repositories and accession number(s) can be found below: https://www.ncbi.nlm.nih.gov/geo/, GSE140692.

## Ethics Statement

The studies involving human participants were reviewed and approved by Ethical Committee of the Department of Medical Sciences, University of Guanajuato. The patients/participants provided their written informed consent to participate in this study.

## Author Contributions

AP-T and GAS-M performed most of the bioinformatics analysis. NF-B assisted in the bioinformatics analysis. DR-R performed most of the molecular biology work. ME and SM generated the microarray data. SZ participated in designing the study and supervising the experimental work and wrote the initial manuscript version. GL designed the study, supervised the experimental work, and wrote the final version of the manuscript. All the authors read and approved the final manuscript version.

## Conflict of Interest

The authors declare that the research was conducted in the absence of any commercial or financial relationships that could be construed as a potential conflict of interest.

## Footnotes

1 www.ncbi.nlm.nih.gov/geo/

2 david.ncifcrf.gov

## Abbreviations

AA: arachidonic acid
AMM: adult Mexican men cohort
BMI: body mass index
CpG: 5 ′ -CG-3 ′ dinucleotide
DGLA: dihomo- γ-linolenic acid
dmCpG: differentially methylated CpG
DNAm: DNA methylation
EA: elaidic acid
EDA: eicosadienoic acid
FA: fatty acid
FS: fasting state
MUFA: monounsaturated fatty acid
N: normoweight subject
OA: oleic acid
Ob: obese subject
Ow: overweight subject
PA: palmitic acid
PS: postprandial state
PUFA: polyunsaturated fatty acid
SFA: saturated fatty acid.
