# Supplementary material for: Distinct Associations of BMI and Fatty Acids With DNA Methylation in Fasting and Postprandial States in Men
Source: Front Genet. 2021 May 7;12:665769. doi: 10.3389/fgene.2021.665769 (PMC8138173; doi:10.3389/fgene.2021.665769)
Supplement: Supplementary file 7 [file Presentation_1.PPTX]

## Slide 1
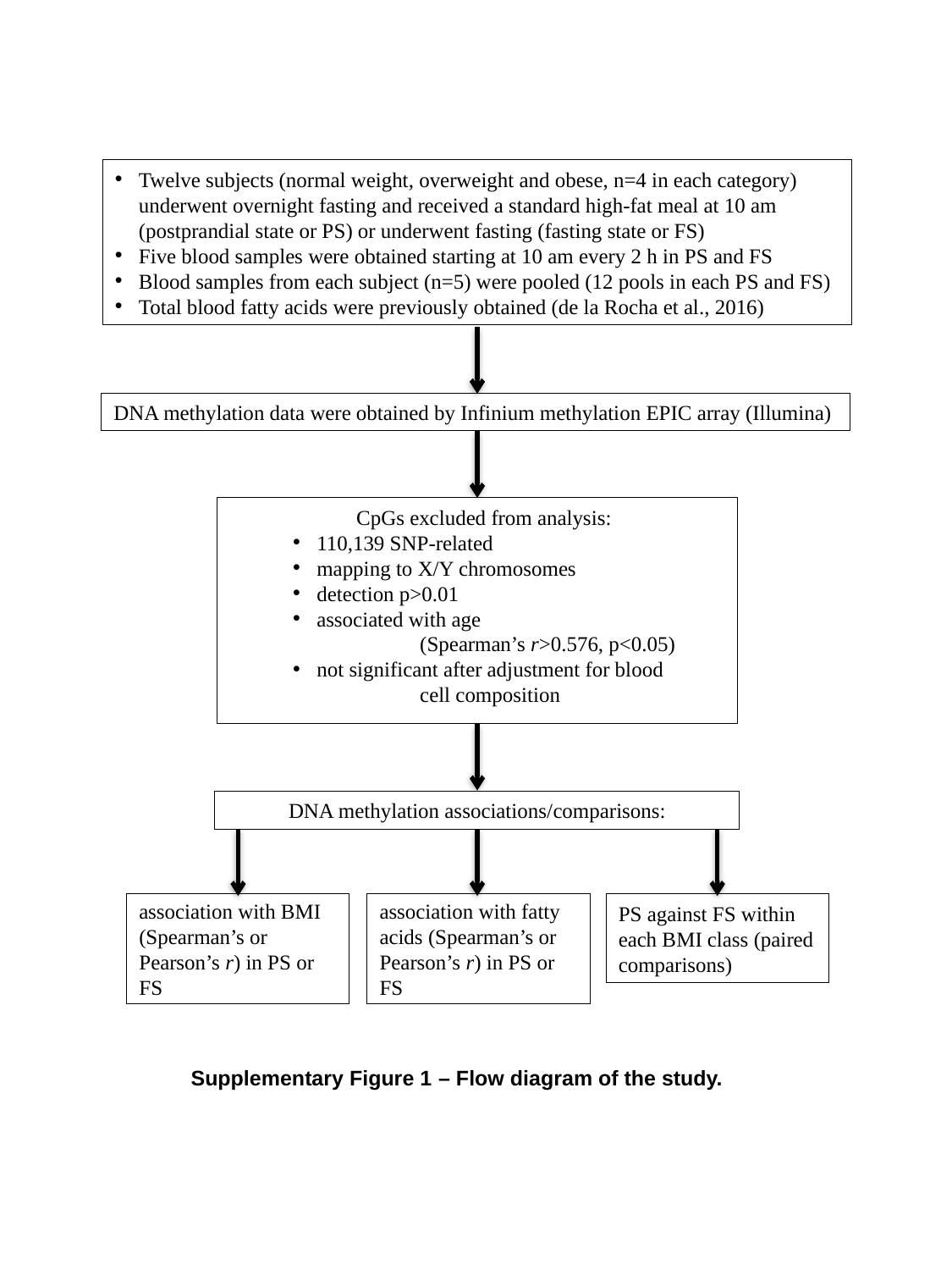

Twelve subjects (normal weight, overweight and obese, n=4 in each category) underwent overnight fasting and received a standard high-fat meal at 10 am (postprandial state or PS) or underwent fasting (fasting state or FS)
Five blood samples were obtained starting at 10 am every 2 h in PS and FS
Blood samples from each subject (n=5) were pooled (12 pools in each PS and FS)
Total blood fatty acids were previously obtained (de la Rocha et al., 2016)
DNA methylation data were obtained by Infinium methylation EPIC array (Illumina)
	CpGs excluded from analysis:
110,139 SNP-related
mapping to X/Y chromosomes
detection p>0.01
associated with age
	(Spearman’s r>0.576, p<0.05)
not significant after adjustment for blood
	cell composition
DNA methylation associations/comparisons:
association with BMI (Spearman’s or Pearson’s r) in PS or FS
association with fatty acids (Spearman’s or Pearson’s r) in PS or FS
PS against FS within each BMI class (paired comparisons)
Supplementary Figure 1 – Flow diagram of the study.
