# Supplementary material for: Distinct Associations of BMI and Fatty Acids With DNA Methylation in Fasting and Postprandial States in Men
Source: Front Genet. 2021 May 7;12:665769. doi: 10.3389/fgene.2021.665769 (PMC8138173; doi:10.3389/fgene.2021.665769)
Supplement: Supplementary file 8 [file Presentation_2.PPTX]

## Slide 1
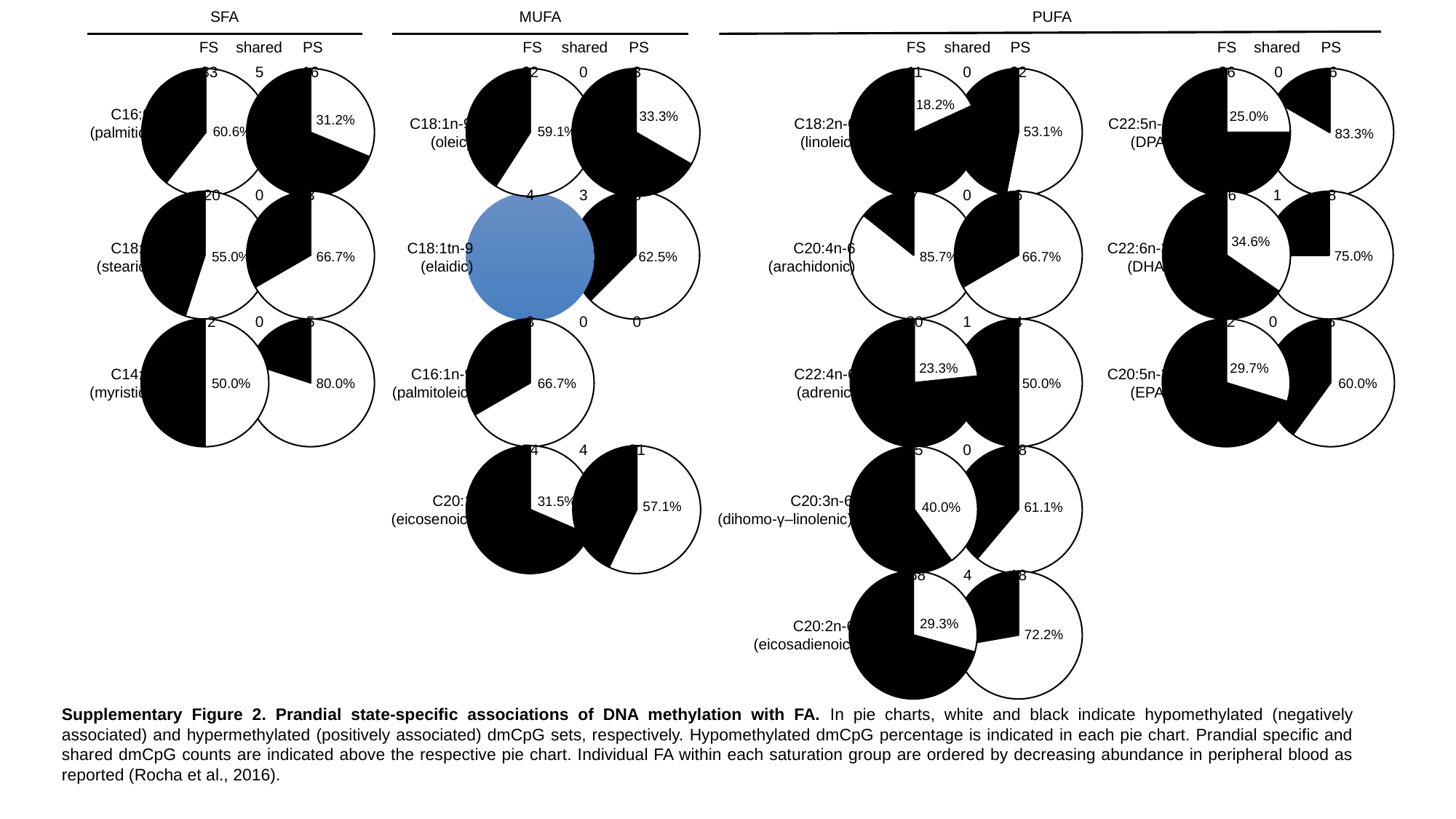

SFA
MUFA
 PUFA
 FS
shared
 PS
 FS
shared
 PS
 FS
shared
 PS
 FS
shared
 PS
33
5
16
22
0
3
11
0
32
36
0
6
### Chart
| Category | |
|---|---|
### Chart
| Category | |
|---|---|
### Chart
| Category | |
|---|---|
### Chart
| Category | |
|---|---|
### Chart
| Category | |
|---|---|
### Chart
| Category | |
|---|---|
### Chart
| Category | |
|---|---|
### Chart
| Category | |
|---|---|18.2%
C16:0
(palmitic)
25.0%
33.3%
31.2%
C18:1n-9
(oleic)
C18:2n-6
(linoleic)
C22:5n-3
(DPA)
60.6%
59.1%
53.1%
83.3%
20
0
3
4
3
8
7
0
6
26
1
8
### Chart
| Category | |
|---|---|
### Chart
| Category | |
|---|---|
### Chart
| Category | |
|---|---|
### Chart
| Category | |
|---|---|
### Chart
| Category | |
|---|---|
### Chart
| Category | |
|---|---|
### Chart
| Category | |
|---|---|
### Chart
| Category | |
|---|---|34.6%
C18:0
(stearic)
C18:1tn-9
(elaidic)
C20:4n-6
(arachidonic)
C22:6n-3
(DHA)
75.0%
55.0%
66.7%
62.5%
85.7%
66.7%
2
0
5
3
0
0
30
1
4
42
0
5
### Chart
| Category | |
|---|---|
### Chart
| Category | |
|---|---|
### Chart
| Category | |
|---|---|
### Chart
| Category | |
|---|---|
### Chart
| Category | |
|---|---|
### Chart
| Category | |
|---|---|
### Chart
| Category | |
|---|---|23.3%
29.7%
C14:0
(myristic)
C16:1n-9
(palmitoleic)
C22:4n-6
(adrenic)
C20:5n-3
(EPA)
50.0%
80.0%
66.7%
50.0%
60.0%
54
4
21
15
0
18
### Chart
| Category | |
|---|---|
### Chart
| Category | |
|---|---|
### Chart
| Category | |
|---|---|
### Chart
| Category | |
|---|---|C20:1
(eicosenoic)
C20:3n-6
(dihomo-γ–linolenic)
31.5%
57.1%
40.0%
61.1%
58
4
18
### Chart
| Category | |
|---|---|
### Chart
| Category | |
|---|---|29.3%
C20:2n-6
(eicosadienoic)
72.2%
Supplementary Figure 2. Prandial state-specific associations of DNA methylation with FA. In pie charts, white and black indicate hypomethylated (negatively associated) and hypermethylated (positively associated) dmCpG sets, respectively. Hypomethylated dmCpG percentage is indicated in each pie chart. Prandial specific and shared dmCpG counts are indicated above the respective pie chart. Individual FA within each saturation group are ordered by decreasing abundance in peripheral blood as reported (Rocha et al., 2016).
