# Supplementary material for: Distinct Associations of BMI and Fatty Acids With DNA Methylation in Fasting and Postprandial States in Men
Source: Front Genet. 2021 May 7;12:665769. doi: 10.3389/fgene.2021.665769 (PMC8138173; doi:10.3389/fgene.2021.665769)
Supplement: Supplementary file 9 [file Presentation_3.PPTX]

## Slide 1
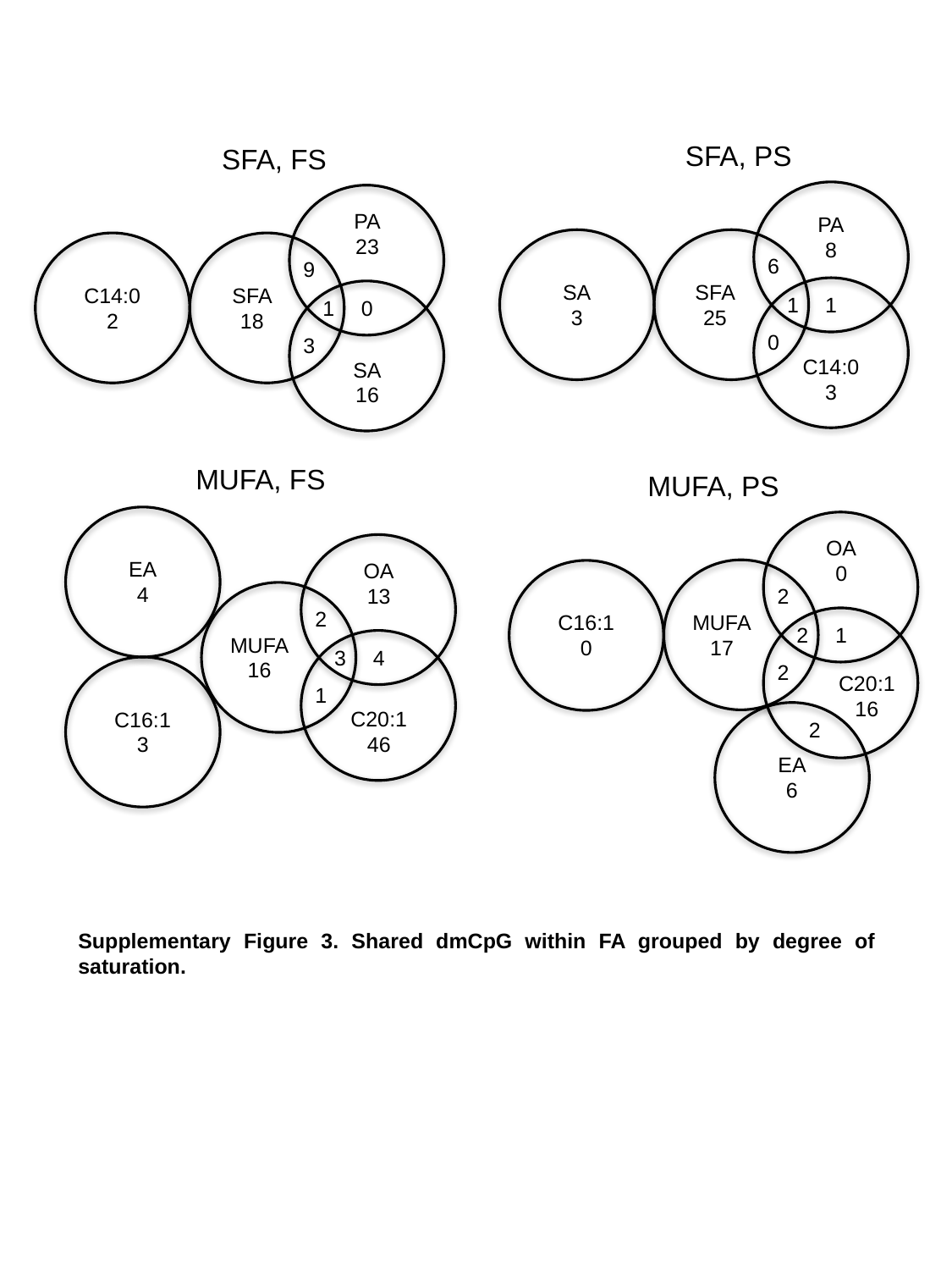

SFA, PS
SFA, FS
PA
23
PA
8
6
9
SA
3
SFA
25
C14:0
2
SFA
18
1
1
1
0
0
3
C14:0
3
SA
16
MUFA, FS
MUFA, PS
OA
0
EA
4
OA
13
2
2
MUFA
17
C16:1
0
2
1
MUFA
16
3
4
2
C20:1
16
1
C20:1
46
C16:1
3
2
EA
6
Supplementary Figure 3. Shared dmCpG within FA grouped by degree of saturation.

## Slide 2
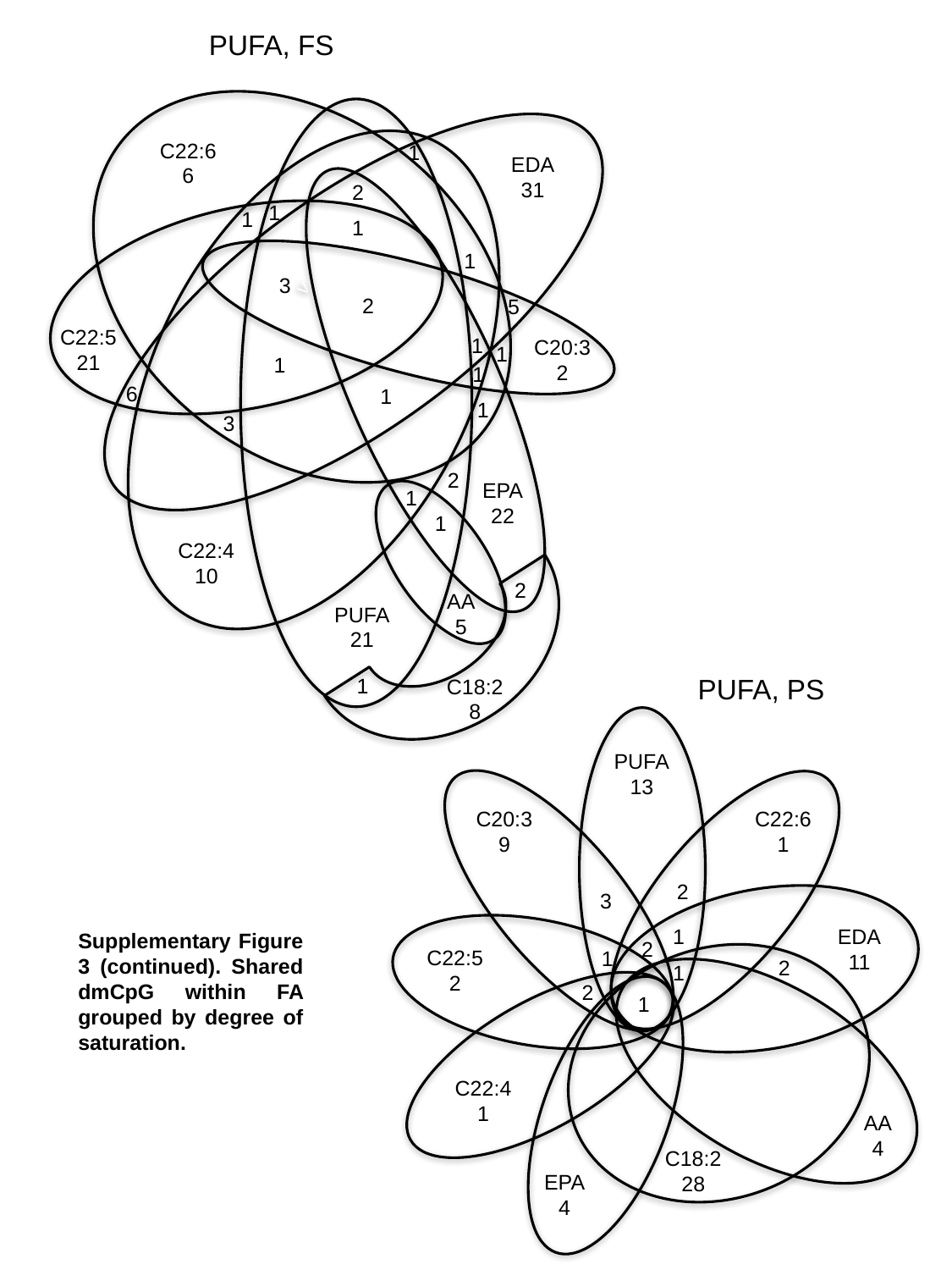

PUFA, FS
v
C22:6
6
1
EDA
31
2
1
1
1
1
3
2
5
C22:5
21
1
C20:3
2
1
1
1
6
1
1
3
2
EPA
22
1
1
C22:4
10
2
AA
5
PUFA
21
PUFA, PS
1
C18:2
8
PUFA
13
C20:3
9
C22:6
1
2
3
1
EDA
11
Supplementary Figure 3 (continued). Shared dmCpG within FA grouped by degree of saturation.
2
C22:5
2
1
2
1
2
1
C22:4
1
AA
4
C18:2
28
EPA
4
