# Supplementary material for: Distinct Associations of BMI and Fatty Acids With DNA Methylation in Fasting and Postprandial States in Men
Source: Front Genet. 2021 May 7;12:665769. doi: 10.3389/fgene.2021.665769 (PMC8138173; doi:10.3389/fgene.2021.665769)
Supplement: Supplementary file 10 [file Presentation_4.PPTX]

## Slide 1
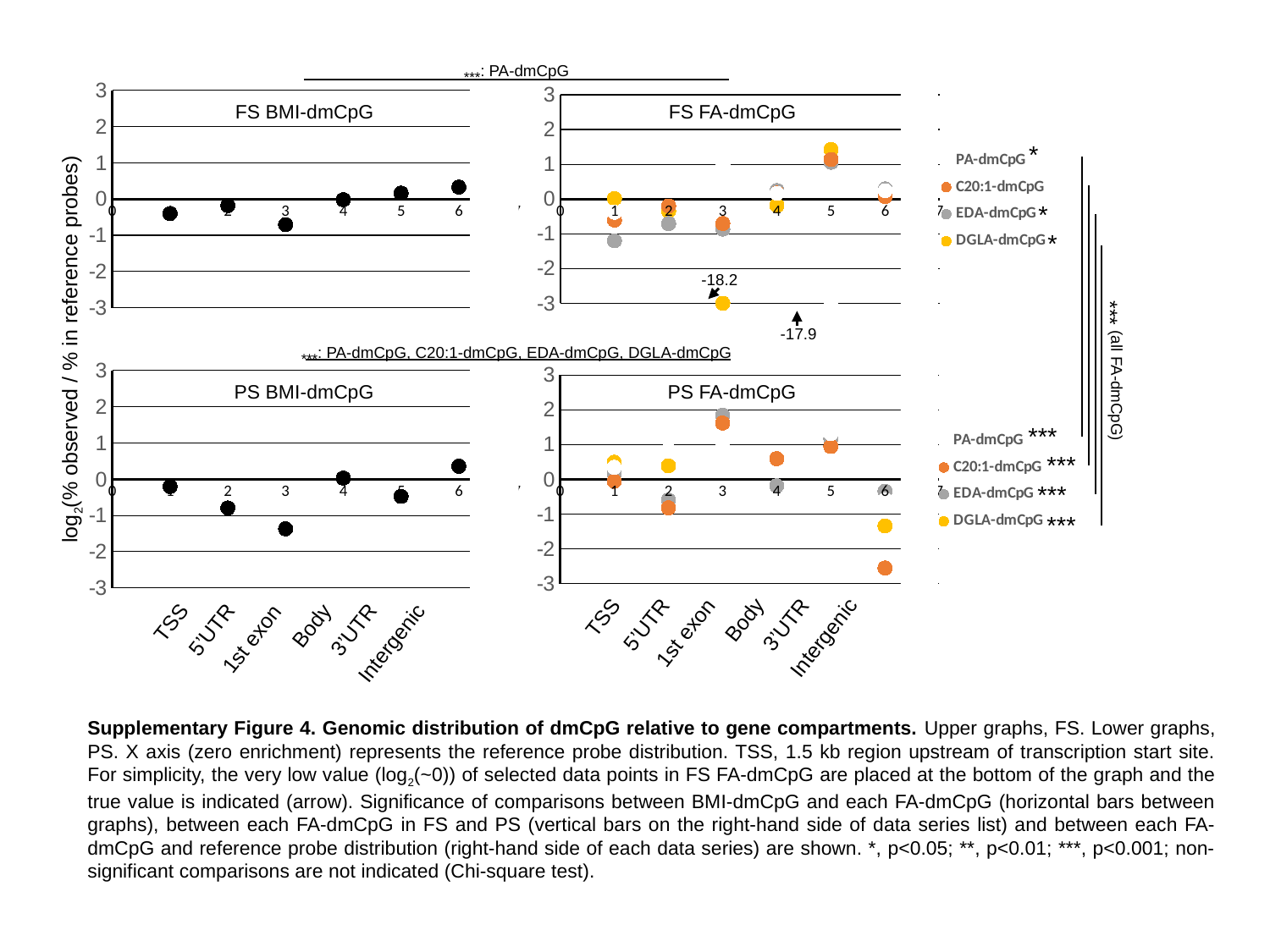

***: PA-dmCpG
### Chart
| Category | FS |
|---|---|
### Chart
| Category | PA-dmCpG | C20:1-dmCpG | EDA-dmCpG | DGLA-dmCpG |
|---|---|---|---|---|
FS FA-dmCpG
FS BMI-dmCpG
*
*
*
-18.2
***
-17.9
***: PA-dmCpG, C20:1-dmCpG, EDA-dmCpG, DGLA-dmCpG
log2(% observed / % in reference probes)
### Chart
| Category | PS |
|---|---|
### Chart
| Category | PA-dmCpG | C20:1-dmCpG | EDA-dmCpG | DGLA-dmCpG |
|---|---|---|---|---|(all FA-dmCpG)
PS BMI-dmCpG
PS FA-dmCpG
***
***
***
***
TSS
Body
TSS
5’UTR
3’UTR
Body
5’UTR
3’UTR
1st exon
Intergenic
1st exon
Intergenic
Supplementary Figure 4. Genomic distribution of dmCpG relative to gene compartments. Upper graphs, FS. Lower graphs, PS. X axis (zero enrichment) represents the reference probe distribution. TSS, 1.5 kb region upstream of transcription start site. For simplicity, the very low value (log2(~0)) of selected data points in FS FA-dmCpG are placed at the bottom of the graph and the true value is indicated (arrow). Significance of comparisons between BMI-dmCpG and each FA-dmCpG (horizontal bars between graphs), between each FA-dmCpG in FS and PS (vertical bars on the right-hand side of data series list) and between each FA-dmCpG and reference probe distribution (right-hand side of each data series) are shown. *, p<0.05; **, p<0.01; ***, p<0.001; non-significant comparisons are not indicated (Chi-square test).

## Slide 2
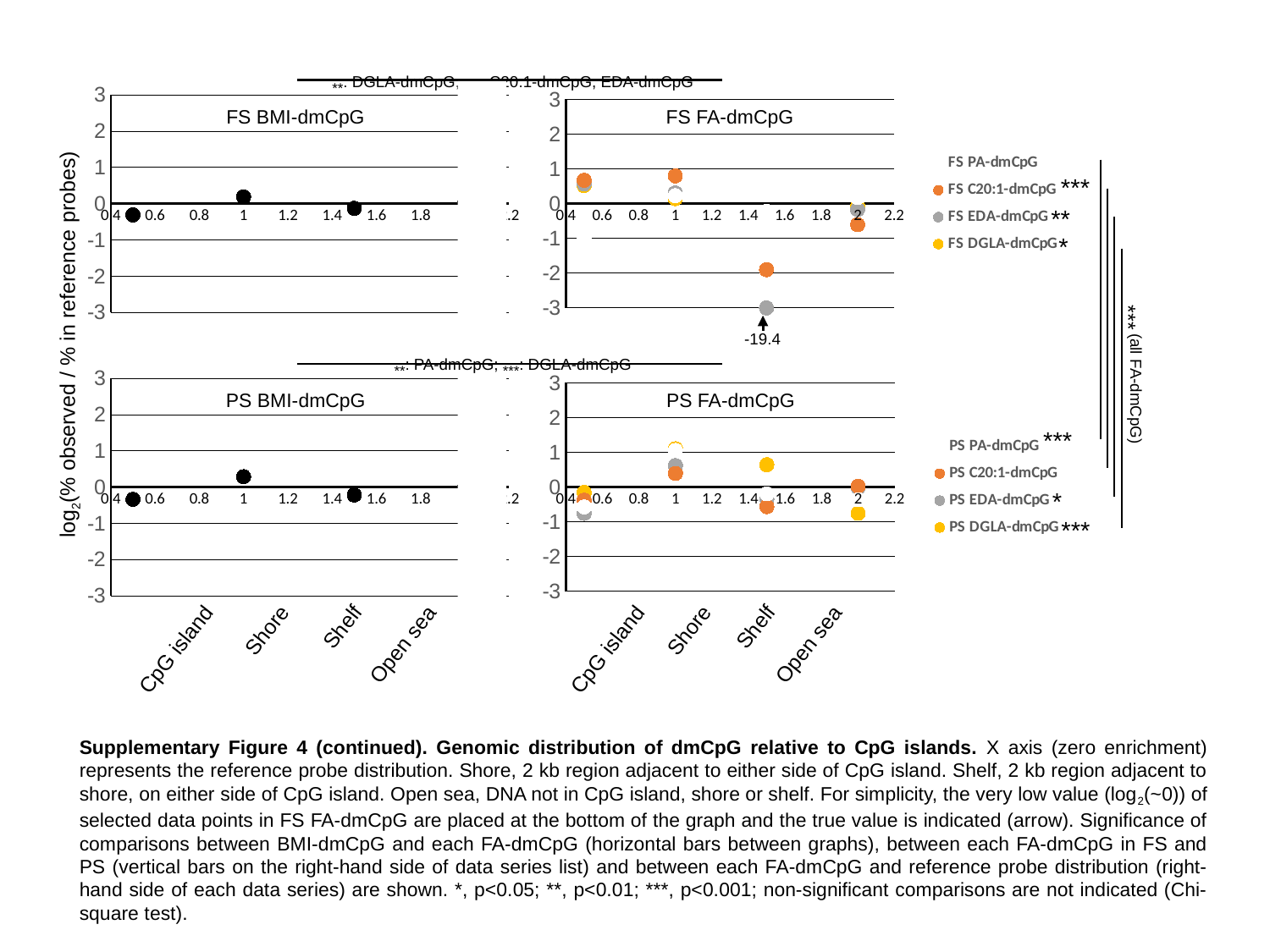

**: DGLA-dmCpG; ***: C20:1-dmCpG, EDA-dmCpG
### Chart
| Category | FS |
|---|---|
### Chart
| Category | FS PA-dmCpG | FS C20:1-dmCpG | FS EDA-dmCpG | FS DGLA-dmCpG |
|---|---|---|---|---|
FS FA-dmCpG
FS BMI-dmCpG
***
**
*
***
 **: PA-dmCpG; ***: DGLA-dmCpG
-19.4
log2(% observed / % in reference probes)
### Chart
| Category | PS |
|---|---|
### Chart
| Category | PS PA-dmCpG | PS C20:1-dmCpG | PS EDA-dmCpG | PS DGLA-dmCpG |
|---|---|---|---|---|(all FA-dmCpG)
PS BMI-dmCpG
PS FA-dmCpG
***
*
***
Shelf
Shelf
Shore
Shore
Open sea
Open sea
CpG island
CpG island
Supplementary Figure 4 (continued). Genomic distribution of dmCpG relative to CpG islands. X axis (zero enrichment) represents the reference probe distribution. Shore, 2 kb region adjacent to either side of CpG island. Shelf, 2 kb region adjacent to shore, on either side of CpG island. Open sea, DNA not in CpG island, shore or shelf. For simplicity, the very low value (log2(~0)) of selected data points in FS FA-dmCpG are placed at the bottom of the graph and the true value is indicated (arrow). Significance of comparisons between BMI-dmCpG and each FA-dmCpG (horizontal bars between graphs), between each FA-dmCpG in FS and PS (vertical bars on the right-hand side of data series list) and between each FA-dmCpG and reference probe distribution (right-hand side of each data series) are shown. *, p<0.05; **, p<0.01; ***, p<0.001; non-significant comparisons are not indicated (Chi-square test).

## Slide 3
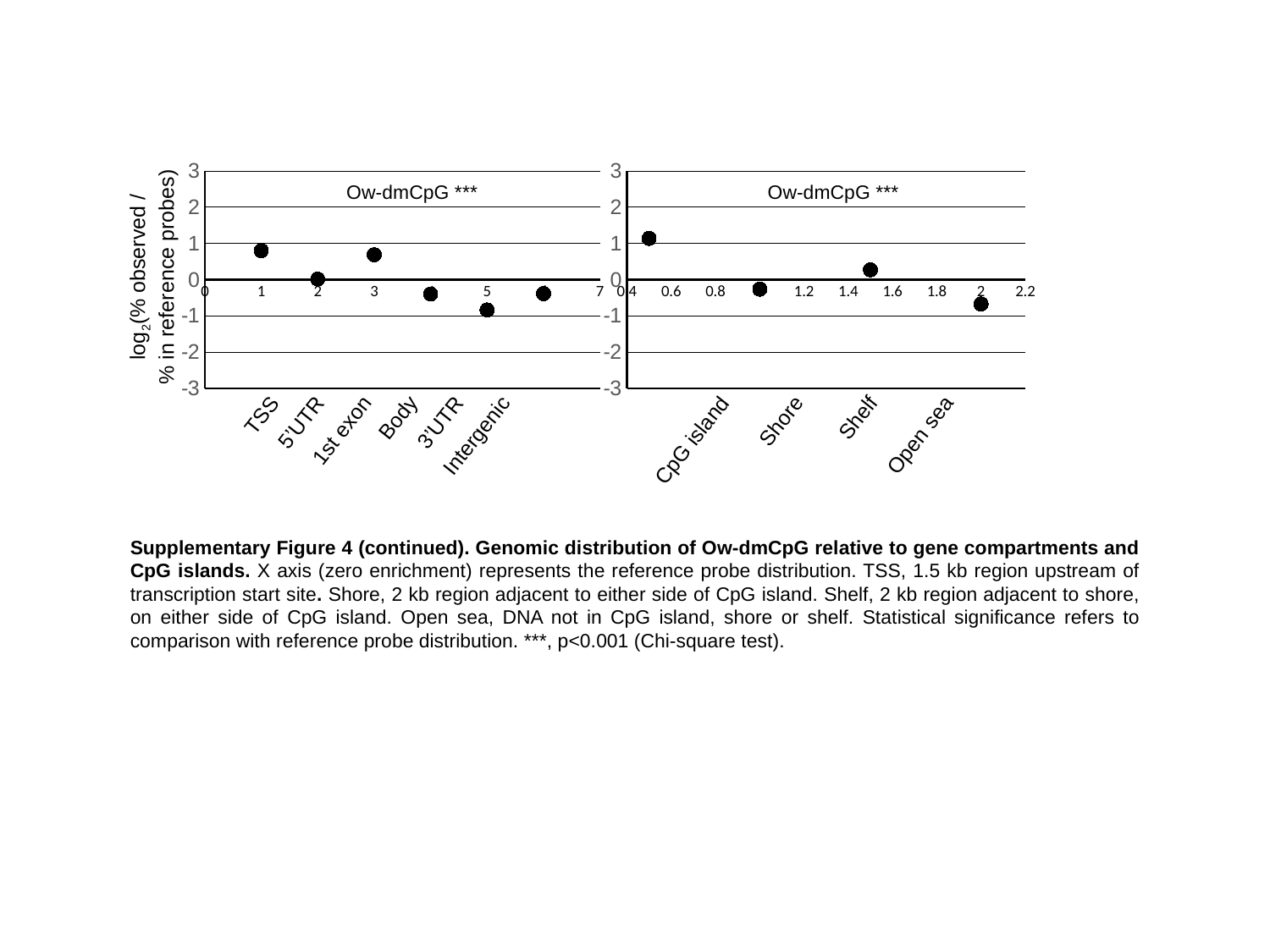

### Chart
| Category | Ow |
|---|---|
### Chart
| Category | Ow |
|---|---|Ow-dmCpG ***
Ow-dmCpG ***
log2(% observed /
% in reference probes)
TSS
Body
Shelf
Shore
5’UTR
3’UTR
1st exon
Open sea
Intergenic
CpG island
Supplementary Figure 4 (continued). Genomic distribution of Ow-dmCpG relative to gene compartments and CpG islands. X axis (zero enrichment) represents the reference probe distribution. TSS, 1.5 kb region upstream of transcription start site. Shore, 2 kb region adjacent to either side of CpG island. Shelf, 2 kb region adjacent to shore, on either side of CpG island. Open sea, DNA not in CpG island, shore or shelf. Statistical significance refers to comparison with reference probe distribution. ***, p<0.001 (Chi-square test).
