# Supplementary material for: Distinct Associations of BMI and Fatty Acids With DNA Methylation in Fasting and Postprandial States in Men
Source: Front Genet. 2021 May 7;12:665769. doi: 10.3389/fgene.2021.665769 (PMC8138173; doi:10.3389/fgene.2021.665769)
Supplement: Supplementary file 11 [file Presentation_5.PPTX]

## Slide 1
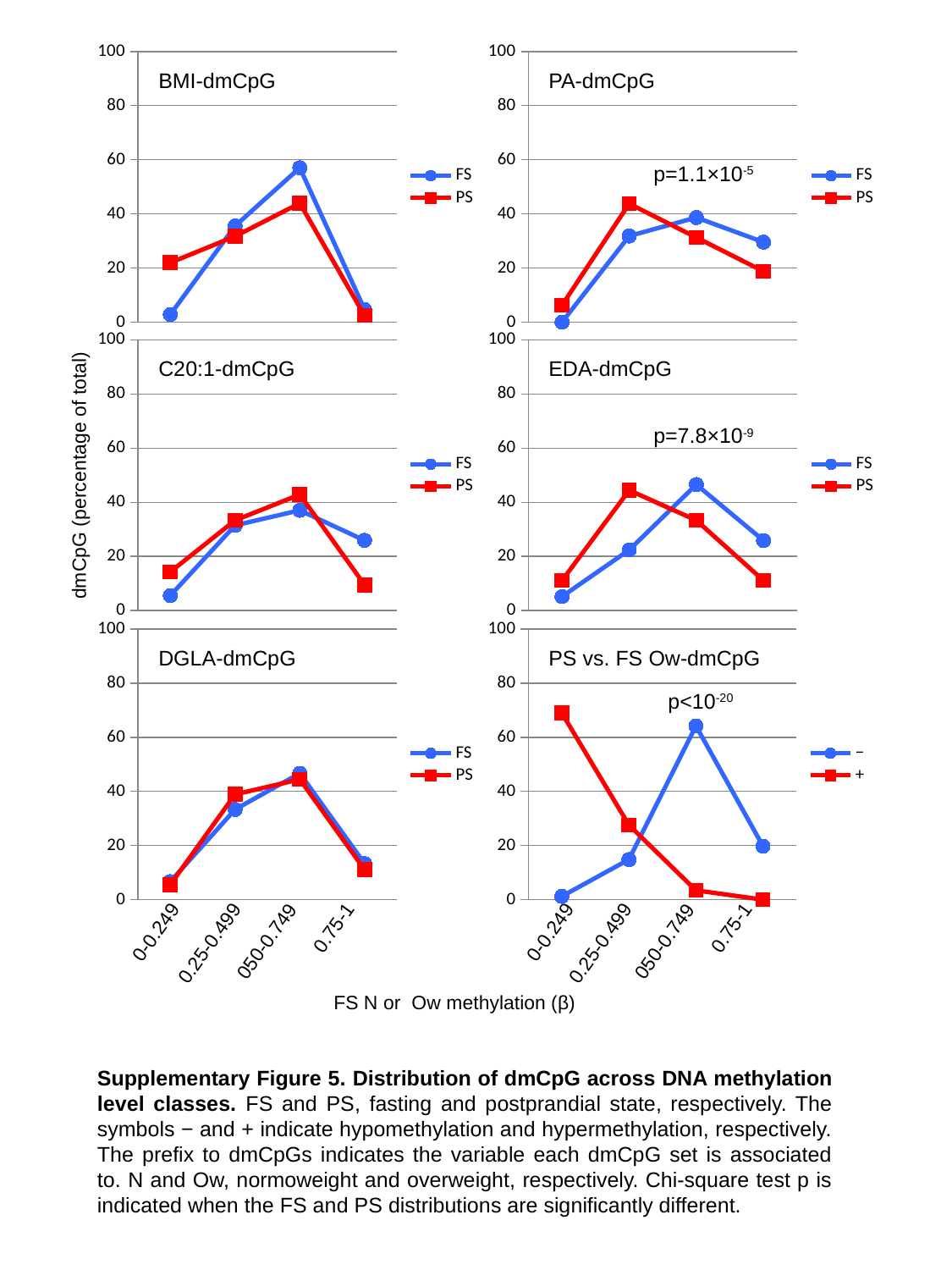

### Chart
| Category | FS | PS |
|---|---|---|
### Chart
| Category | FS | PS |
|---|---|---|BMI-dmCpG
PA-dmCpG
p=1.1×10^-5^
### Chart
| Category | FS | PS |
|---|---|---|
| 0-0.249 | 5.555555555555554 | 14.28571428571428 |
| 0.25-0.499 | 31.48148148148148 | 33.33333333333333 |
| 0.5-0.749 | 37.03703703703704 | 42.85714285714284 |
| 0.75-1 | 25.92592592592592 | 9.523809523809524 |
### Chart
| Category | FS | PS |
|---|---|---|
| 0-0.249 | 5.172413793103448 | 11.11111111111111 |
| 0.25-0.499 | 22.41379310344828 | 44.44444444444443 |
| 0.5-0.749 | 46.55172413793102 | 33.33333333333333 |
| 0.75-1 | 25.86206896551724 | 11.11111111111111 |C20:1-dmCpG
EDA-dmCpG
p=7.8×10^-9^
dmCpG (percentage of total)
### Chart
| Category | FS | PS |
|---|---|---|
| 0-0.249 | 6.666666666666667 | 5.555555555555554 |
| 0.25-0.499 | 33.33333333333333 | 38.8888888888889 |
| 0.5-0.749 | 46.66666666666664 | 44.44444444444443 |
| 0.75-1 | 13.33333333333333 | 11.11111111111111 |
### Chart
| Category | − | + |
|---|---|---|DGLA-dmCpG
PS vs. FS Ow-dmCpG
p<10-20
0.75-1
0.75-1
0-0.249
0-0.249
050-0.749
050-0.749
0.25-0.499
0.25-0.499
FS N or Ow methylation (β)
Supplementary Figure 5. Distribution of dmCpG across DNA methylation level classes. FS and PS, fasting and postprandial state, respectively. The symbols − and + indicate hypomethylation and hypermethylation, respectively. The prefix to dmCpGs indicates the variable each dmCpG set is associated to. N and Ow, normoweight and overweight, respectively. Chi-square test p is indicated when the FS and PS distributions are significantly different.
